# AMPK is required for recovery from metabolic stress induced by ultrasound microbubble treatment

**DOI:** 10.1016/j.isci.2022.105883

**Published:** 2022-12-28

**Authors:** Louis Lo, Oro Uchenunu, Roberto J. Botelho, Costin N. Antonescu, Raffi Karshafian

**Affiliations:** 1 Department of Chemistry and Biology, Toronto Metropolitan University, Toronto, ON M5B 2K3, Canada; 2 Graduate Program in Molecular Science, Toronto Metropolitan University, Toronto, ON M5B 2K3, Canada; 3 Faculty of Medicine and Health Sciences, Division of Experimental Medicine, McGill University, Montreal, QC H3A 0G4, Canada; 4 Department of Physics, Toronto Metropolitan University, Toronto, ON M5B 2K3, Canada; 5 Institute for Biomedical Engineering, Science and Technology (iBEST), a partnership between Ryerson University and St. Michael’s Hospital, Toronto, ON M5B 1W8, Canada; 6 Keenan Research Centre for Biomedical Science of St. Michael’s Hospital, Toronto, ON M5B 1W8, Canada

**Keywords:** Biological sciences, Cellular physiology, Biotechnology, Cell

## Abstract

Ultrasound-stimulated microbubble (USMB) treatment is a promising strategy for cancer therapy. USMB promotes drug delivery by sonoporation and enhanced endocytosis, and also impairs cell viability. However, USMB elicits heterogeneous effects on cell viability, with apparently minimal effects on a subset of cells. This suggests that mechanisms of adaptation following USMB allow some cells to survive and/or proliferate. Herein, we used several triple negative breast cancer cells to identify the molecular mechanisms of adaptation to USMB-induced stress. We found that USMB alters steady-state levels of amino acids, glycolytic intermediates, and citric acid cycle intermediates, suggesting that USMB imposes metabolic stress on cells. USMB treatment acutely reduces ATP levels and stimulates the phosphorylation and activation of AMP-activated protein kinase (AMPK). AMPK is required to restore ATP levels and support cell proliferation post-USMB treatment. These results suggest that AMPK and metabolic perturbations are likely determinants of the antineoplastic efficacy of USMB treatment.

## Highlights

• Ultrasound and microbubble (USMB) treatment alters levels of intracellular metabolites
• AMPK is activated to restore cellular ATP following USMB treatment
• AMPK is required to support recovery and cell proliferation following USMB treatment

## Graphical abstract

**Figure:**
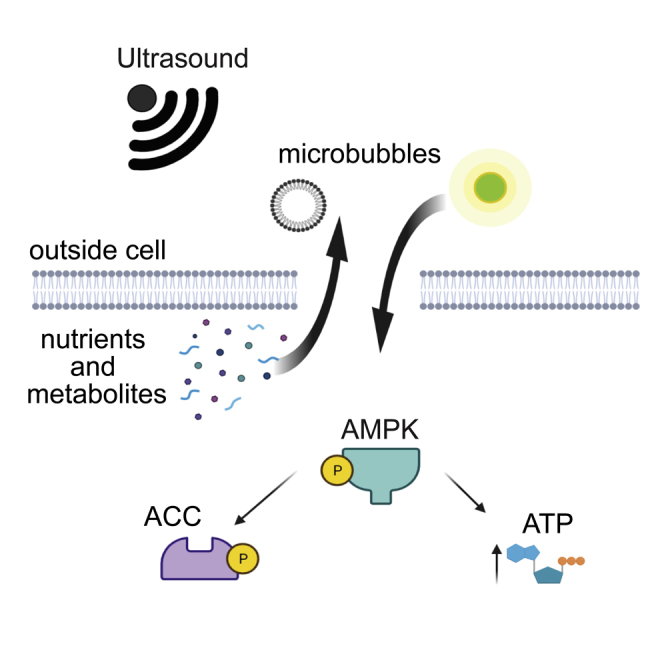

## Introduction

The application of therapeutic ultrasound waves on biological tissues can induce a range of changes to cell and tissue physiology. These biological effects of ultrasound are amplified in the presence of microbubbles (MBs) which are micron-sized, gas-filled spherical structures stabilized by a polymeric shell of lipids and proteins.^1^^,^^2^ Combinations of MBs and ultrasound are being investigated for clinical applications including those aiming to improve targeted drug delivery in cancer^3^^,^^4^^,^^5^ and targeted delivery through the blood-brain barrier.^6^^,^^7^ The effectiveness of ultrasound-stimulated microbubble (USMB) treatment as a targeted delivery strategy in cell culture models was demonstrated for various agents including chemotherapy, gold nanoparticles, genetic material, and antibiotics.^4^^,^^8^^,^^9^^,^^10^ The effects of USMB treatment are in large part mediated by enhanced uptake of extracellular material through pore formation on the plasma membrane, referred to as sonoporation,^11^^,^^12^ and enhancement of endocytic uptake by several pathways.^3^^,^^13^^,^^14^^,^^15^ However, cells exposed to USMB alone may exhibit a variety of cellular effects including activation of diverse signaling cascades which regulate cell survival, apoptosis, and cellular adaptation.^16^^,^^17^^,^^18^ Although these studies demonstrate that targeted USMB treatment alone can induce cell death in a small volume within a tumor, the factors that contribute to the survival of a subset of cancer cells following this treatment are poorly understood. As such, understanding the mechanisms that cells engage to adapt to USMB-induced cell stress is critical.

A less appreciated outcome of sonoporation is that this phenomenon also enhances the diffusion of cellular material to the extracellular milieu.^19^ Consistent with this, we previously reported the loss of molecules such as cytosolic GFP-tagged proteins from the cell following USMB treatment.^19^ This suggests that a range of intracellular molecules may also be released during sonoporation, leading to their depletion from within the cell.^19^ We thus hypothesized that USMB treatment may lead to the loss of key metabolites including nucleotides, glucose, glycolytic intermediates, and amino acids, and that loss of these may result in metabolic and signaling perturbations that can impact cell fate.^20^^,^^21^^,^^22^

Cells respond to changes in available nutrients^21^^,^^22^^,^^23^^,^^24^^,^^25^^,^^26^ by adjusting their metabolic pathways to maintain homeostasis.^27^^,^^28^ Under periods of high nutrient availability, cellular signaling pathways enable anabolism and cell growth.^24^ In contrast, under periods of nutrient insufficiency, key biochemical pathways enable catabolism and restoration of metabolic homeostasis.^25^ For instance, AMP-activated protein kinase (AMPK) is a key cellular sensor of metabolic stress.^20^^,^^21^ AMPK consist of a catalytic α subunit, and regulatory β and γ subunits.^29^ Cells experiencing nutrient depletion also often exhibit an increase in ADP and AMP levels relative to ATP. AMP/ADP competes for ATP to bind to some cystathionine β-synthetase domains on the γ subunits of AMPK, eliciting a conformational change that leads to enhanced phosphorylation of Thr172 on the α subunit by LKB1, leading to AMPK activation.^29^ Nutrient stress in the form of reduced glucose availability also activates AMPK in a manner independent of ATP levels.^30^ AMPK is also activated in response to increases in intracellular calcium and mechanical stress transduced by E-cadherin, both requiring AMPK phosphorylation by LKB1 or CaMKK.^21^^,^^29^^,^^31^^,^^32^ Once activated, AMPK restores cellular metabolic balance, in general by decreasing anabolic processes while stimulating catabolic pathways. For instance, AMPK decreases lipogenesis while stimulating fatty acid oxidation via phosphorylation and inactivation of acetyl-CoA-carboxylase (ACC).^33^ Moreover, AMPK can inactivate mTORC1 signaling thus resulting in the reduction of protein synthesis.^21^

In this work, we postulated that USMB triggers metabolic stress and activates AMPK, and that AMPK may consequently reprogram cells to support viability and/or proliferation of a subset of cells following USMB treatment. To test this, we employed USMB treament on MDA-MB-231, SUM149PT, and BT-20 triple negative breast cancer cell lines. We used several approaches to reveal that USMB treatment leads to decreased intracellular levels of various metabolites while concurrently stimulating AMPK activity. We then found that AMPK is necessary for cells to recover from this USMB-mediated metabolic insult and proliferate following USMB treatment. This suggests that co-administration of AMPK inhibitors during USMB may be an effective anti-tumor strategy.

## Results

### USMB treatment leads to the influx of extracellular material

**Figure 1:**
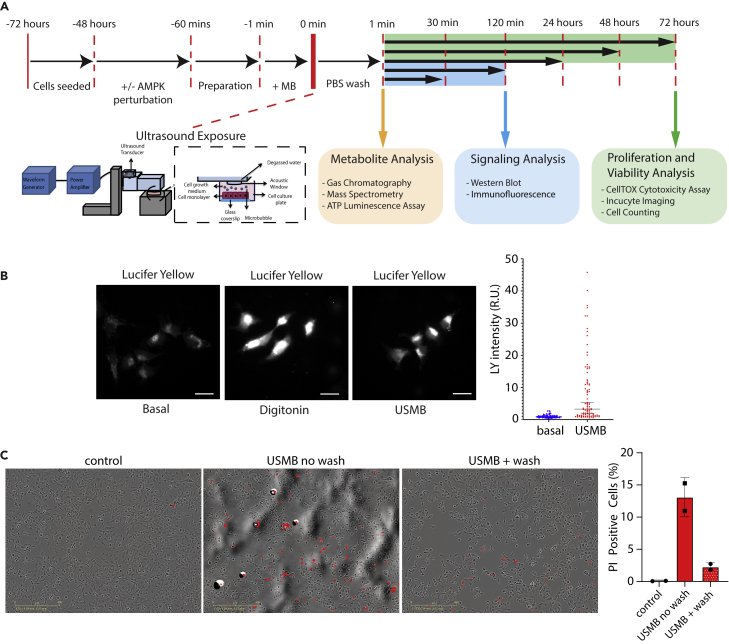
Design of the study (A) MDA-MB-231, SUM149PT or BT-20 cells were prepared and treated with USMB using a calibrated acoustic exposure platform that was developed to deliver ultrasound pulses, as was previously described.^15^ See STAR Methods for additional details of USMB treatment. Numerous assays were performed following USMB treatment over a 72 h period as indicated, including metabolite analysis (Figures 2 and 4), Signaling Analysis (Figures 3 and 5) and proliferation and viability assays (Figures 6 and 7). (B) MDA-MB-231 cells were subject to USMB treatment (USMB) or 2 μg/mL digitonin for 5 min in the continuous presence of Lucifer Yellow Potassium Salt (125 μg/mL), as indicated, to label (reversibly or irreversibly) sonoporated cells. Shown (left panels) are representative images obtained by widefield epifluorescence microscopy, scale bar = 100 μm. Also shown (right panels) is the quantification of Lucifer Yellow fluorescence, depicting fluorescence measured in individual cells (individual points) and median (horizontal bar) with 95% confidence interval (CI). (C) MDA-MB-231 cells were treated with USMB and then only subsequently incubated with Propidium Iodide (PI), which thus identifies irreversibly sonoporated cells. For some cell monolayer samples, no wash was performed after USMB treatment (USMB no wash), whereas the USMB + wash condition involved several washes to remove non-viable cells before PI staining and imaging; the latter is the standard experimental protocol used throughout this study. Shown (left panels) are representative images as overlays of phase contrast and PI fluorescence. Scale bar, 400 μm. Also shown (right panel) is the quantification of PI-positive cells, showing the mean ± SD of 2 independent experiments.

Considering that our previous study showed that USMB treatment causes efflux of GFP-tagged proteins from cells,^19^ we set out to investigate the effects of USMB on the steady-state metabolome of surviving cells. To achieve this, we applied USMB treatment on MDA-MB-231 breast cancer cells using a custom-built ultrasound transducer system (Figure 1A). To establish the optimal conditions for USMB treatment of MDA-MB-231 cells, we monitored the influx of plasma-membrane impermeable dye, Lucifer Yellow by confocal microscopy (Figure 1B). USMB exposure increased the average fluorescence intensity of Lucifer Yellow per cell. We observed ∼80% of USMB-treated cells with Lucifer Yellow intensity (median = 3.267) higher than the upper 95% confidence interval of the mean of the untreated cells (Basal, Upper 95% CI of mean = 1.1039) (Figure 1B, right), indicating that this USMB treatment induced sonoporation. As a positive control, we perforated cell membrane with digitonin which resulted in significant increase in fluorescence intensity compared to untreated cells (Figure 1B).

To validate that irreversibly sonoporated and thus non-viable cells were excluded from our experimental analysis and subsequent downstream assays, we labeled cells with propidium iodide (PI) following USMB (Figure 1C, left). Of note, this PI is added following USMB treatment and, therefore, labels cells that are non-viable following USMB treatment. In contrast, LY treatment was done during USMB, and thus labels both reversibly and irreversibly sonoporated cells. In cells imaged without washing cell monolayers in PBS, we observe ∼15% of cells positive for PI following USMB. However, following several washes in PBS, the proportion of PI-positive cells decreased substantially (Figure 1C, right). Hence, all subsequent experiments were done by washing cells in PBS following USMB treatment to remove non-viable cells, thus ensuring that we focus our study on initially viable, reversibly sonoporated cells during our analyses.

### USMB treatment alters cellular metabolite levels

**Figure 2:**
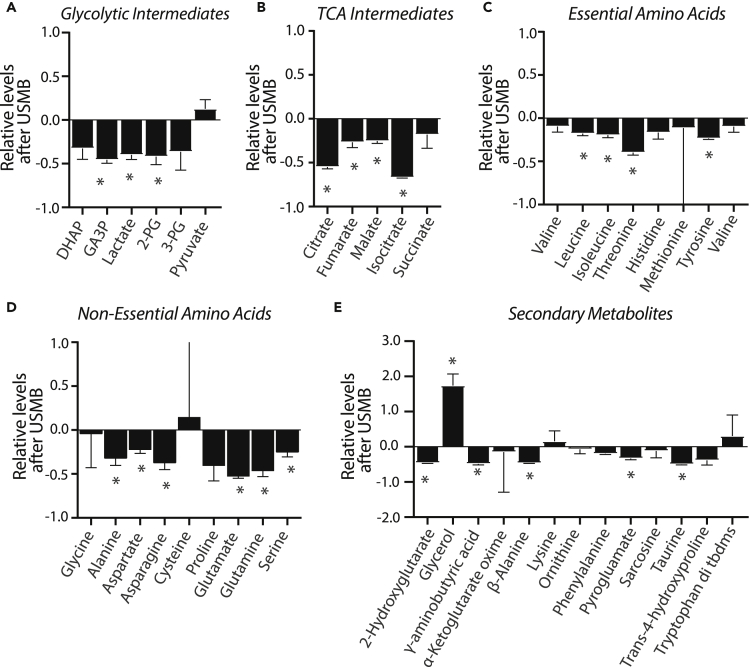
USMB impacts a broad range of cellular levels of metabolites and amino acids Levels of indicated metabolites in MDA-MB-231 cells treated with USMB were determined by gas chromatography-mass spectrometry (GC/MS). Data are shown as a mean ± SD (n = 3) expressed as levels detected in USMB-treated samples relative to untreated control; Relative levels = ([USMB_treated]-[control])/[control]) for each metabolite. Statistical analysis was performed by unpaired t-tests (comparing control versus USMB-treated samples) for each metabolite, ∗, p< 0.05. DHAP, dihydroxyacetone phosphate; GA3P Glyceraldehyde-3-phosphate; 2-PG, 2-phosphoglycerate; 3-PG, 3-phosphoglycerate.

We hypothesized that USMB causes a loss of cytosolic contents such as glycolytic and citric acid cycle (TCA) metabolites and amino acids, which are directly involved in numerous aspects of cellular function and survival.^23^ To assess this, intracellular steady-state levels of select metabolites were quantified post-USMB treatment. USMB treatment reduced the levels of many glycolytic and citric acid cycle intermediates as well as essential and non-essential amino acids (Figures 2A–2E). Notably, the extent of loss of various metabolites following USMB treatment varied, suggesting that following initial depletion of certain metabolites from cells after USMB treatment, cataplerosis and/or anaplerosis may partly replenish some metabolites at the time of the assay. For example, although USMB caused a significant decrease in glyceraldehyde-3-phosphate (GA3P) and 3-phosphoglycerol (3-PG), the impact of USMB on dihydroxyacetone phosphate (DHAP) and 2-phosphoglycerol (2-PG) appeared to be less pronounced (Figure 2B). Similarly, the TCA intermediates citrate, fumarate, and malate were significantly reduced in USMB-treated cells, whereas changes in other TCA metabolites such as succinate were not observed following USMB treatment (Figure 2B).

The intracellular levels of essential amino acids (leucine, isoleucine, and threonine), non-essential amino acids derived from citric acid cycle intermediates (glutamine, glutamate, and aspartate), and other non-essential amino acids (alanine, asparagine, tyrosine, and serine), were all also significantly reduced by USMB treatment (Figures 2C and 2D). As observed for some glycolytic and TCA intermediates, not all amino acids levels were uniformly affected by USMB treatment, as the levels of glycine, cysteine and proline did not exhibit appreciable changes following USMB treatment (Figures 2C and 2D). A number of secondary metabolites (2-hydroxyglutarate, GABA, β-alanine, ornithine, phenylamine, and taurine) were also significantly reduced relative to control, untreated cells (Figure 2E). We also observed a large accumulation of glycerol following USMB treatment; however, this is likely because of the remnants of microbubble debris where glycerol is a key ingredient of the DEFINITY microbubble shell composition (Lantheus Medical Imaging, DEFINITY, MSDS, 10/4/2015). Overall, these results indicate that USMB-induced sonoporation of MDA-MB-231 cells has a broad effect to significantly reduce the intracellular levels of a diverse set of metabolites. This, in turn, suggests that cells exposed to USMB treatment may suffer significant energetic stress owing to loss of key metabolites and nutrients.

### AMPK is activated following USMB treatment

**Figure 3:**
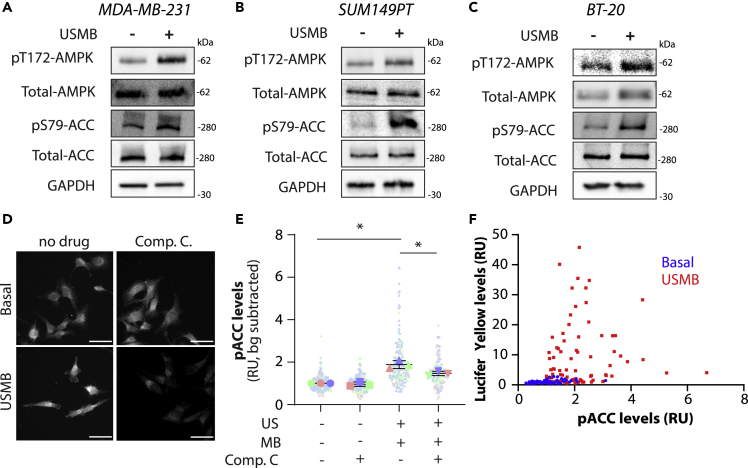
USMB triggers AMPK activation (A–C) MDA-MB-231, SUM149PT, and BT-20 cells were treated with USMB or left untreated (basal). Whole cell lysates were prepared 30 min after USMB treatment and were analyzed by Western blotting with depicted antibodies. (D and E) MDA-MB-231 cells were fixed, permeabilized, and stained for endogenous pS79-ACC, 30 min after USMB treatment. Some samples were also pre-treated with 10 μM compound C for 1 h before USMB treatment, as indicated. Shown in (D) are representative images obtained by widefield epifluorescence microscopy, scale bar, 100 μm. Shown in (E) is the quantification of immunofluorescence intensity; data is represented as normalized fluorescence (relative units, R.U.) values in individual cells (small circles), average cell fluorescence intensity in each experiment in each condition (large shapes) and mean of independent experiments (bar) ± SD (whiskers). The experiment was repeated three times independently. Measurements are color-coded by independent experiment. Statistical analysis was performed by one-way ANOVA followed by Tukey’s multiple comparisons test. ∗, p < 0.05. (F) A similar experiment was performed in which MDA-MB-231 were subject to USMB in the presence of Lucifer Yellow Potassium Salt (125 μg/mL), then subject to fixation and staining. Shown is the quantification of immunofluorescence intensity of phospho-ACC and Lucifer Yellow for individual MDA-MB-231 cells treated with USMB as indicated.

Given our observation that USMB reduced glycolytic intermediates and amino acids in surviving cells, we hypothesized that USMB-induced sonoporation induces AMPK activation. To determine whether USMB treatment triggered AMPK activation, we probed for phosphorylation of AMPK (pT172-AMPK) and acetyl CoA-carboxylase (pS79-ACC), a substrate of AMPK, in three triple negative breast cancer cell models. Western blotting of whole cell lysates revealed that USMB treatment resulted in greater phosphorylation of pT172-AMPK and pS79-ACC in MDA-MB-231, SUM149PT, and BT-20 cells (Figures 3A–3C). We complemented this approach with immunofluorescence microscopy to detect pS79-ACC after USMB treatment in single MDA-MB-231 cells (Figure 3D). We found that pS79-ACC was increased following USMB treatment in comparison to the untreated control (Figure 3E). Furthermore, pharmacological inhibition of AMPK using 10 μM Compound C for 1 h before USMB reduced pS79-ACC fluorescence, indicating that the increase in ACC phosphorylation in USMB-treated conditions indeed reflected enhanced AMPK activation (Figure 3E).

The broad distribution of pS79-ACC staining intensity in cells after USMB treatment suggests a heterogeneous response to USMB treatment (Figure 3E). If the heterogeneity in AMPK activation following USMB reflects heterogeneity in the extent of sonoporation, and thus the extent of loss of cellular metabolites, then the levels of sonoporation and AMPK activation should correlate in individual cells. To this end, we examined both pS79-ACC staining intensity along with the uptake of Lucifer Yellow (a marker of sonoporation, Figure 2A) in response to USMB treatment. The majority of USMB-treated cells that exhibited elevated pS79-ACC levels also had higher Lucifer Yellow fluorescence intensity (mean = 8.190) (Figure 3F), indicating that sonoporation and AMPK activation co-occur.

### AMPK is required for the restoration of intracellular ATP following USMB treatment

**Figure 4:**
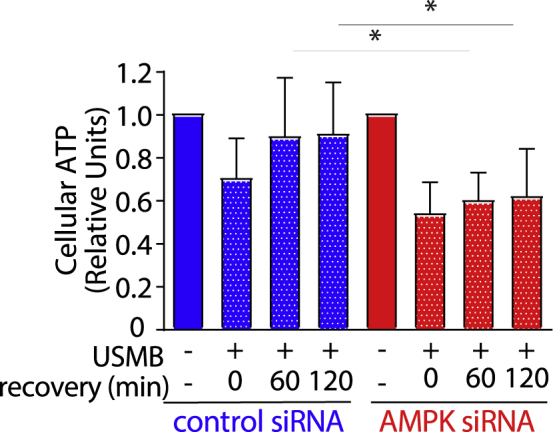
Recovery of cellular ATP levels following USMB treatment requires AMPK MDA-MB-231 cells were treated with control (non-targeting) or AMPK siRNA. Following transfection, cells were treated with USMB as indicated, which was then followed by a recovery period as shown. Cell samples were then subject to a luminescence-based assay to measure ATP levels. ATP measurements were normalized to total protein and also normalized to the control (no USMB) condition. Shown are the mean ATP values ±SD, (n = 7). Statistical analysis was performed by one-way ANOVA followed by Tukey’s multiple comparisons test. ∗, p < 0.05.

Because we observed a general decrease across measured metabolites following USMB treatment, we hypothesized that AMPK activation plays a key role in restoring energy and metabolite balance following USMB-induced sonoporation. To test this, we measured ATP levels in the cells before and after USMB treatment in MDA-MB-231 cells. This revealed that USMB treatment induces an acute loss of cytosolic ATP (after <5 min of USMB treatment, Figure 4). Importantly, cytosolic ATP levels were restored 1 h after USMB treatment (Figure 4, blue bars). To determine if AMPK plays a role in recovery of ATP levels following USMB treatment, we used short interfering RNA (siRNA) to silence the catalytic α subunit of AMPK (both α1 and α2 isoforms) in parallel experiments and quantified intracellular ATP levels following USMB treatment. In AMPK-depleted cells, we observed similar reduction in intracellular ATP levels immediately following USMB treatment as in control siRNA treated cells. However, in contrast with control siRNA treated cells, there was no appreciable recovery of ATP levels for up to 2 h after treatment (Figure 4, red bars). These observations imply that USMB triggers a reduction in intracellular ATP levels, and that AMPK is required for recovery of ATP levels.

### AMPK is required for cellular recovery following USMB treatment

**Figure 5:**
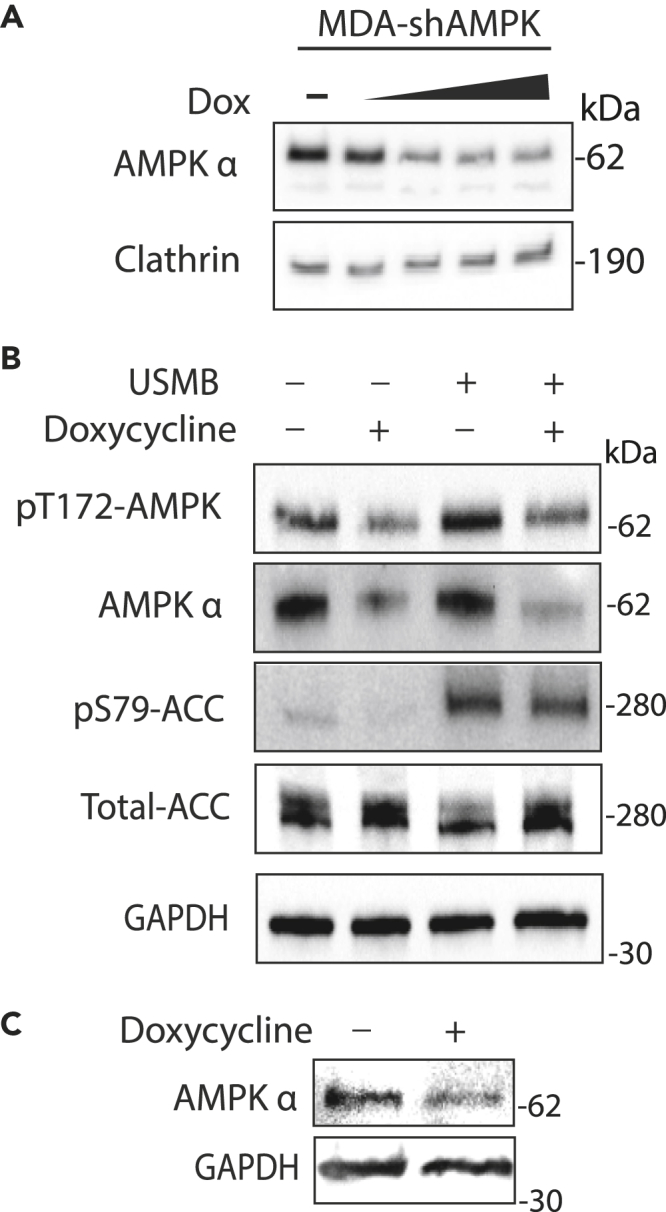
Inducible expression of AMPK shRNA achieves effective silencing of AMPK activity (A) Expression of AMPK shRNA in MDA-shAMPK was induced by incubation of 0.1–5 μM doxycycline for 48 h. Shown are representative western blots of whole cell lysates probed with antibodies specific for AMPK α1/2 or clathrin (loading control). (B) Expression of AMPK shRNA in MDA-shAMPK was induced by incubation of 3 μM doxycycline for 48 h, as indicated. Cells were then also then treated with USMB, as indicated, and whole cell lysates were prepared 30 min after USMB treatment. Shown are representative western blots with antibodies as indicated. (C) Expression of AMPK in SUM149PT-shAMPK cells following incubation of 3 μM doxycycline for 48 h, as indicated. Shown are representative western blots with antibodies as indicated.

To study the effects of USMB and the role of AMPK on cell survival and growth following USMB treatment, we generated MDA-MB-231 and SUM149PT cells with doxycycline-inducible expression of short hairpin RNA (shRNA) targeting AMPK (MDA-shAMPK and SUM149PT-shAMPK henceforth). As expected, doxycycline treatment for 48 h resulted in dose-dependent silencing of AMPKα1/2 in MDA-shAMPK cells (Figure 5A). Treatment with 3 μM doxycycline for 48 h resulted in approximately 50–80% loss of the AMPKα1/2 subunit in MDA-shAMPK (Figure 5B) and SUM149PT-shAMPK (Figure 5C) cells. Importantly, in MDA-shAMPK, USMB-treated cells depleted of AMPK by doxycycline treatment exhibited reduced pT172-AMPK and pS79-ACC compared to cells treated with USMB and expressing AMPK (without doxycycline treatment; Figure 5B). Collectively, these results demonstrate that AMPK levels and/or activity was effectively suppressed in MDA-shAMPK and SUM149PT-shAMPK cells following addition of doxycycline.

**Figure 6:**
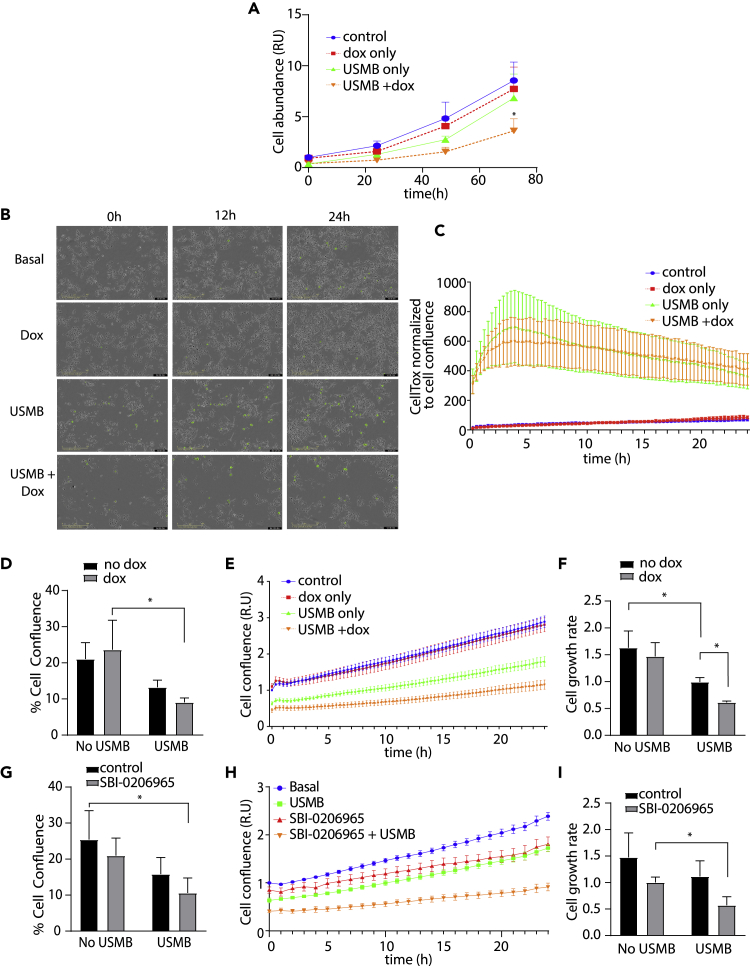
AMPK is required for MDA-MB-231 cell growth following USMB treatment (A–F) MDA-shAMPK cells were treated ±3 μM doxycycline for 48 h (dox) followed by USMB treatment as indicated. (A) Cell counting assay was performed every 24 h; measurements were normalized to the untreated control condition and shown as mean ± SD (n = 5). Statistical analysis was performed by two-way ANOVA with Tukey’s multiple comparison test. ∗, p < 0.05, relative to corresponding timepoint in control (no doxycycline, no USMB treatment) condition. (B–F) Following doxycycline (dox) and USMB treatments, cells were subjected to time-lapse imaging with sequential phase contrast and CellTox Green fluorescence image acquisition. (B) Representative images from time-lapse recording of MDA-shAMPK cells treated with a solution containing CellTox Green. Representative images shown as overlay of phase contrast and CellTox Green fluorescence. Scale bar, 400 μm. (C) Quantification of CellTox Green positive objects (cells) normalized to cell confluence shown mean ± SEM of n = 3 independent experiments. (D–F) Quantification of cell confluence determined from phase contrast images of MDA-shAMPK cells treated as indicated. Shown in (D) is the percent confluence at t = 0 min following USMB, normalized to untreated control measured ±SD (n = 3). Statistical analysis was performed by two-way ANOVA with Sidak’s multiple comparison. ∗, p<0.05. Shown in (E) is the relative confluence at regular intervals over 24 h following USMB treatment, normalized to untreated control ±SD (n = 3). Shown in (F) is the result of determination of the rate of cell growth (slope of values of each individual experiment in E) shown as mean ± SD (n = 3). Statistical analysis was performed by two-way ANOVA with Sidak’s multiple comparison. ∗, p < 0.05. (G–I) MDA-MB-231 cells were treated with USMB and treated ± SBI-0206965 (1 μM) for 24 h as indicated, then subject to time-lapse phase contrast imaging and quantification as in D–F. Shown in (G) is the percent confluence at t = 0 min following USMB, normalized to untreated control measured ±SD (n = 3). Statistical analysis was performed by two-way ANOVA with Sidak’s multiple comparison. ∗, p<0.05. Shown in (H) is the relative confluence at regular intervals over 24 h following USMB treatment, normalized to untreated control ±SD (n = 3). Shown in (I) is the result of determination of the rate of cell growth (slope of values of each individual experiment in H) shown as mean ± SD (n = 3). Statistical analysis was performed by two-way ANOVA with Sidak’s multiple comparison. ∗, p < 0.05.

So far, our observations show that USMB treatment elicits a reduction in the levels of many metabolites, including ATP. Cells that survive USMB treatment recover ATP levels by 2 h post-treatment in an AMPK-dependent manner. We thus hypothesized that suppressing AMPK would lead to a decrease in cell viability and/or arrest cell proliferation in response to USMB treatment. By counting the number of total cells, we found that in the absence of USMB treatment, MDA-shAMPK cells treated with doxycycline to deplete AMPK exhibited comparable proliferation rates as control cells not treated with doxycycline (Figure 6A, blue and red lines). In contrast, AMPK depletion by doxycycline treatment led to significant reduction of the total number of cells following USMB treatment (Figure 6A, orange line) relative to cells treated with USMB alone (without doxycycline treatment) (Figure 6A, green line). This suggests that AMPK is essential to support cell proliferation and/or viability of MDA-MB-231 cells after USMB treatment.

To further explore the role of AMPK in adaptation to USMB-induced stress, we simultaneously monitored MDA-MB-231 cell viability and proliferation using an automated environmentally controlled microscope system (Figure 6B and Video S1). Cell death was detected in real-time by labeling cells with CellTox Green and by normalizing against confluence to account for possible differences in cell abundance when assessing the fraction of non-viable cells. Of note, these dead (non-viable) cells are distinct from the irreversibly sonoporated cells that become non-viable immediately on USMB treatment that are washed away before this assay. Instead, the detection of CellTox Green-positive cells allows identification of cells that were initially reversibly sonoporated but undergo delayed cell death following USMB treatment. Consistent with past studies,^34^^,^^35^^,^^36^ we found that MDA-MB-231 cells treated with USMB exhibited a rapid increase in CellTox Green-positive cells (starting as early as 1h following USMB) in comparison to non-USMB exposed cells, indicating loss of cell viability (Figures 6B and 6C orange and green lines). Following USMB treatment, MDA-shAMPK cells displayed a similar level of acute loss of cell viability by this method, whether or not they were treated with doxycycline to deplete AMPK (Figure 6D). These observations indicate that MDA-MB-231 cells do not rely on AMPK to enhance cell viability following USMB treatment either acutely, as seen immediately (<5 min) after USMB treatment (Figure 6D), as well as over a 24h period following USMB treatment (Figure 6C).

Video S1. Time-lapse recordings of MDA-shAMPK cells treated with a solution containing CellTox Green, related to Figure 6B MDA-shAMPK cells were treated ± 3 μM doxycycline for 48 h (dox) followed by USMB treatment as indicated in Figure 6. Video shows the following in sequence: “Basal” (no USMB or doxycycline), “Dox only” (doxycycline treated condition resulting in AMPK silencing), “USMB only” (no doxycycline), “USBM + dox” (doxycycline treated sample followed by USMB treatment).

We next examined the long-term effects of USMB treatment, analogously to experiments in Figure 6A. MDA-shAMPK cells treated or not with doxycycline started at similar confluence (20%) and increased at similar rates over 24 h (Figures 6E and 6F). As expected, given the immediate, acute reduction in cell viability due to irreversible sonoporation, USMB treatment significantly reduced initial confluence, which was similar whether or not cells were treated with doxycycline to suppress AMPK expression (Figures 6D and 6E). However, over 24 h, the increase in confluence on USMB treatment was attenuated by AMPK depletion (Figures 6E and 6F), indicating that AMPK is needed to support cell proliferation following USMB treatment. To complement the results obtained with shRNA silencing of AMPK in MDA-MB-231 cells, we next examined the effect of pharmacological inhibition of AMPK with SBI-0206965^37^ on the response of these cells to USMB treatment. Consistent with our observations with AMPK shRNA, cells treated with SBI-0206965 and exposed to USMB exhibited reduced cell proliferation over 24 h compared to cells treated with USMB alone (Figures 6G–6I).

**Figure 7:**
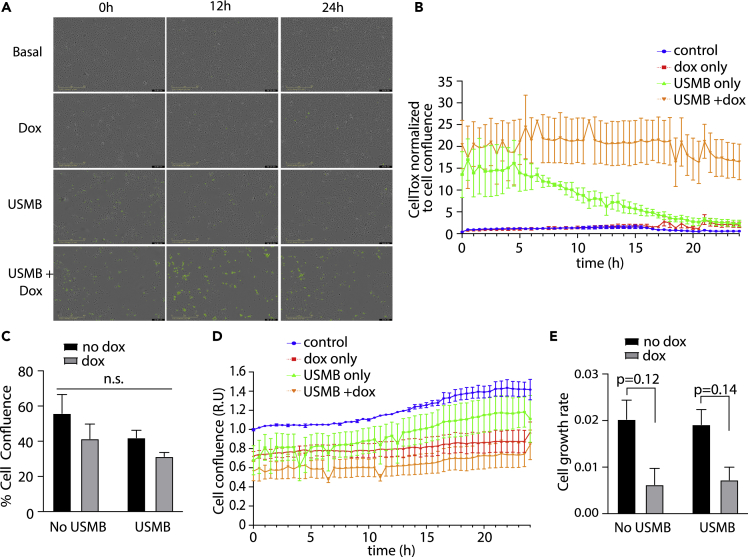
AMPK is required for SUM149PT cell survival following USMB treatment SUM149PT-shAMPK cells were treated ±3 μM doxycycline for 48 h (dox) followed by USMB treatment as indicated. (A) Representative images from time-lapse recording of SUM149PT-shAMPK cells treated with a solution containing CellTox Green. Representative images shown as overlay of phase contrast and CellTox Green fluorescence. Scale bar, 400 μm. (B) Quantification of total CellTox Green positive objects (cells) relative to the cell confluence, shown as mean ± SEM of n = 3 independent experiments. Statistical analysis was performed by two-way ANOVA followed by Tukey post-test. All measurements of cell viability for the USMB + dox condition from 7 to 24h after USMB are significantly different (p < 0.05) compared to USMB only treated cells. (C–E) Quantification of cell confluence determined from phase contrast images of SUM149PT-shAMPK cells treated as indicated. Shown in (C) is the percent confluence at t = 0 min following USMB, normalized to untreated control measured ±SD (n = 3). Statistical analysis was performed by two-way ANOVA with Sidak’s multiple comparison. Shown in (D) is the relative confluence at regular intervals over 24 h following USMB treatment, normalized to untreated control, shown as the mean ± SEM of n = 3 independent experiments. (E) Rate of cell growth (slope of values of each individual experiment in (D) shown as the mean ± SEM of n = 3 independent experiments.

To expand these observations for a role for AMPK in cells following USMB treatment, we examined SUM149PT cells. We again examined cell death and cell confluence in SUM149PT-shAMPK cells in response to USMB treatment (Figures 7A–7C). USMB treatment resulted in a rapid reduction in cell viability as detected by CellTox Green positive cells, which in cells expressing AMPK resolved after ∼5–10 h post-USMB, likely as a result of non-viable cells detaching from the adherent surface (Figures 7A and 7B). In contrast, SUM149PT-shAMPK that had been treated with doxycycline to achieve AMPK silencing exhibited sustained loss of cell viability throughout the 24 h observation period, indicating that these cells underwent ongoing loss of viability (Figure 7B). In contrast with these robust effects on cell viability that are sustained subsequent to the initial effects of USMB treatment, the cell confluence immediately following USMB treatment (Figure 7C) or the rate of cell proliferation measuring by cell confluence was not significantly affected by USMB treatment (Figures 7D and 7E). Together, these observations indicate that AMPK plays a critical role in cellular adaptation and recovery from USMB-induced stress in MDA-MB-231 and SUM149PT cells, though specific aspects of these effects may be cell type specific.

## Discussion

The potential theragnostic applications of ultrasound in combination with microbubbles for cancer is gaining interest as unique cell biological effects of USMB treatment are revealed and characterized. Until now, USMB treatment has been well-established to facilitate drug delivery into cells through sonoporation and enhanced endocytosis mechanisms.^38^ For example, systemic administration of microbubbles in the circulation with targeted ultrasound to the tumor can lead to biological effects in cancer cells in the tumor, including increased cell death or drug uptake.^39^^,^^40^ In addition, direct administration of microbubbles into tumors or intravascular administration of nanobubbles^41^ may further localize the effects of USMB to cancer cells in a tumor.^42^ The focus of USMB treatment for drug delivery has been largely centered on bringing mostly impermeable extracellular materials (drugs or nucleic acids) into the cell through sonoporation as opposed to the cell biological effects associated with loss of intracellular material such as its use to specifically kill target cancer cells. Here, we demonstrate that USMB treatment reduces the levels of many intracellular metabolites, in particular amino acids, and glycolytic and citric acid cycle intermediates. To recover from this insult, MDA-MB-231 and SUM149PT cells rely on AMPK activation. Thus, inhibiting AMPK during USMB treatment may represent a strategy to enhance killing of cancer cells in a tumor.

Our metabolome analysis revealed that USMB treatment causes a reduction in the intracellular levels of a broad range of metabolites. A possible mechanism for this reduction in metabolite levels is the exchange of cytosolic materials from the cell interior to the extracellular milieu. However, the direct loss of metabolites by diffusion seems unlikely to account for all reductions in metabolite levels, such as those of the TCA cycle that should be found within mitochondrial matrix, and thus are less likely to directly efflux from the cell as a result of USMB-induced plasma membrane pores, suggesting instead that anaplerosis and/or cataplerosis contribute to changes in some metabolites following USMB treatment. Although beyond the scope of the current study, future work that may delineate the precise molecular mechanisms underpinning USMB-induced changes in metabolic flux may further reveal the metabolic impact of USMB treatment on cancer cells.

USMB treatment leads to a reduction in ATP levels within the cell, and AMPK is required for restoring cellular ATP within 2 h following USMB treatment (Figure 4). There are several mechanisms by which AMPK may promote metabolic adaptation resulting in replenishment of ATP levels.^20^^,^^43^ For instance, AMPK activation increases glucose uptake and promotes glycolysis, by acutely increasing the transport of glucose via facilitative glucose transporters such as GLUT1^44^ and phosphorylation of phosphofructokinase 2^45^^,^^46^ as well as by transcriptional regulation of various glycolytic enzymes.^45^^,^^46^^,^^47^ Furthermore, ACC activity is suppressed as a result of its phosphorylation by AMPK and its activity can be further abated by reduced citrate concentrations.^48^ Hence, ACC inactivation by AMPK suppresses fatty acid synthesis while increasing fatty acid oxidation,^33^ leading to restoration of ATP levels.^33^ AMPK activation also represses additional metabolic pathways such as hexosamine^49^^,^^50^ and mevalonate biosynthesis.^51^ Future work should strive to define which of these mechanisms primarily drives cellular adaptation to USMB-induced loss of metabolites and ATP.

Our results further indicate that AMPK activation supports cancer cell proliferation. Although many studies report that activation of AMPK, such as with biguanides like metformin, suppresses proliferation of cancer cells,^52^^,^^53^^,^^54^ other studies have shown that in certain contexts, AMPK activation is required to support cell proliferation. In lung cancer driven by oncogenic KRAS mutation, abrogation of AMPK led to substantial impairment of tumor growth, revealing the role of AMPK to promote lysosome expansion and autophagy.^55^ The tumor-promoting functions of AMPK are also associated with AMPK enhancement of glucose transporter expression and glucose transport,^44^ enhanced carbohydrate metabolic capacity supported by heightened glycolytic enzyme expression,^20^^,^^45^^,^^56^ and improved mitochondrial capacity, such as that resulting from AMPK regulation of PGC1α.^20^^,^^57^ The selective requirement for AMPK control of subsets of these functions in MDA-MB-231 and SUM149-PT cells may explain how AMPK promotes cell proliferation or cell survival in each cell line following USMB treatment, respectively. Furthermore, although we are not aware of any AMPK inhibitors available clinically or in trials, our results suggest that once such inhibitors become available, they could be useful in combination therapy with USMB for treatment of certain cancers. Overall, our results reveal that AMPK is important for at least some cancer cells to adapt following USMB treatment, thus supporting cell proliferation and/or survival.

### Limitations of the study

The sonoporation phenomenon has been extensively studied in a number of models; *in vivo* (animals, solid tumors)^58^^,^^59^ and *in vitro* (cultured cancer cells and non-cancer cells).^16^^,^^60^^,^^61^ Although we have shown activation of AMPK following USMB treatment in three TNBC cell types (MBA-MB-231, SUM149PT and BT20), other cell types and cell lines may respond and behave differently when exposed to USMB.^60^ Moreover, the nature and extent of the cell biological effects of USMB may depend on various parameters of ultrasound settings and microbubble properties, such as concentration and formulation of microbubbles, and ultrasound parameters (soundwave, microbubble formulation, duration of treatment), which were previously defined for the cell lines we examined here.

## STAR★Methods

### Key resources table

**Table:** 

REAGENT or RESOURCE	SOURCE	IDENTIFIER
**Antibodies**		

AMPKα Antibody	Cell Signaling Technologies	RRID:AB_330331; Cat#2532
Phospho-AMPKα (Thr172) (40H9) Rabbit mAb	Cell Signaling Technologies	RRID:AB_331250; Cat#2535
Phospho-Acetyl-CoA Carboxylase (Ser79) (D7D11) Rabbit mAb	Cell Signaling Technologies	RRID:AB_2687505; Cat#11818
Acetyl-CoA Carboxylase Antibody	Cell Signaling Technologies	RRID:AB_2219400; Cat#3662
Clathrin Heavy Chain (D3C6) XP® Rabbit mAb	Cell Signaling Technologies	RRID:AB_10828486; Cat#4796
GAPDH (14C10) Rabbit mAb	Cell Signaling Technologies	RRID:AB_561053; Cat#2118
Anti-rabbit IgG, HRP-linked Antibody	Cell Signaling Technologies	RRID:AB_2099233; Cat#7074
Alexa Fluor® 647 AffiniPure Donkey Anti-Rabbit IgG (H+L)	Jackson ImmunoResearch Laboratories Inc.	RRID:AB_2492288; Cat#711-605-152
Alexa Fluor® 488 AffiniPure Donkey Anti-Rabbit IgG (H+L)	Jackson ImmunoResearch Laboratories Inc.	RRID:AB_2313584; Cat#711-545-152

**Chemicals, peptides, and recombinant proteins**		

Dorsomorphin (Compound C), AMP-kinase inhibitor	Abcam	Cat#ab120843
Digitonin	Promega	Cat#G9441
Lucifer Yellow CH, Lithium Salt, 25 mg	ThermoFisher Scientific	Cat#L453
Doxycycline, hydrochloride	Bio Basic	Cat#DB0889
CellTox™ Green Cytotoxicity Assay	Promega	Cat#G8741
SBI-0206965	Cell Signaling Technologies	Cat#29089

**Critical commercial assays**		

Luminescent ATP Detection Assay Kit (ab113849)	abcam	Cat#ab113849

**Experimental models: Cell lines**		

MDA-MB-231	ATCC	Cat#HTB-26
BT-20	ATCC	Cat#HTB-19
SUM149PT	Asterand	Cat#HUMANSUM-0003004

**Oligonucleotides**		

RNA Sequences	Sigma-Aldrich – See Table S1 for sequences	Cat#VC30002

**Software and algorithms**		

Incucyte® Base Analysis Software, Incucyte® Live-Cell Analysis System	Sartorius	N/A

**Other**		

DEFINITY® Vial for (Perflutren Lipid Microsphere) Injectable Suspension	Lantheus	Cat#DE16

### Resource availability

#### Lead contact

Further information and requests for resources should be directed to and will be fulfilled by the lead contact, Costin Antonescu, Toronto Metropolitan University, Canada (cantonescu@torontomu.ca).

#### Materials availability

The plasmid encoding inducible expression of AMPK shRNA (in pSBtet-BP) is available from Addgene. MDA-MB-231 and SUM149PT stably transfected cells with the AMPK shRNA transgene are available upon request.

### Experimental methods and subject details

#### Cell lines

The human breast cancer cell line MDA-MB-231 was obtained from AmericanType Culture Collection, ATCC (HTB-26). MDA-MB-231 cells were cultured in RPMI 1640 media supplemented with 10% fetal bovine serum (FBS, ThermoFisher Scientific) and 5% penicillin/streptomycin (ThermoFisher Scientific) in a 37°C and 5% CO_2_ controlled by an incubator system (ThermoFisher Scientific). Cells were passaged every 3 to 5 days.

The human breast cancer cell line SUM149PT was a kind gift from Dr. Daniel Durocher (Lunenfeld-Tanenbaum Research Institute, Toronto, Canada). SUM149PT cells were cultured in Ham’s F-12 media (Gibco) supplemented with 5% fetal bovine serum (FBS, Gibco), 10 mM HEPES (Sigma-Aldrich), 1ug/mL hydrocortisone (Sigma-Aldrich), 5 μg/mL insulin (Sigma-Aldrich), and 5% penicillin/streptomycin (ThermoFisher Scientific) in a 37°C and 5% CO_2_ controlled by an incubator system (ThermoFisher Scientific). Cells were passaged every 3 to 5 days.

The human breast cancer cell line BT-20 was obtained from AmericanType Culture Collection, ATCC (HTB-19). BT-201 cells were cultured in Minimal Essential Media Earle’s Eagle media supplemented with 10% fetal bovine serum (FBS, ThermoFisher Scientific) and 5% penicillin/streptomycin (ThermoFisher Scientific) in a 37°C and 5% CO_2_ controlled by an incubator system (ThermoFisher Scientific). Cells were passaged every 3 to 5 days.

### Method details

#### siRNA-mediated gene silencing

siRNA silencing of AMPK was performed by synthesizing custom siRNA sequences which were designed to silence expression of both AMPK α1/2 isoforms (Table S1). MDA-MB-231 cells were grown to 40% confluency before being subjected to a single round of siRNA transfection using Lipofectamine RNAiMAX transfection (ThermoFisher Scientific, cat#13778075) performed according to manufacturer’s instructions. Cells were exposed to the transfection mixture for 6 h, allowed to rest for 48 h in fresh culture media before treatment.

#### Generation of MDA-MB-231-shAMPK and SUM149PT-shAMPK stable cell lines

To generate stable MDA-MB-231 cells and SUM149PT cells that conditionally silenced AMPK, we designed a sleeping beauty transposon system containing a stable expression of a BFP-reporter gene and a doxycycline inducible gene^62^ to encode expression of a short hairpin sequence that can target both isoforms of the catalytic subunit (α1, α2) AMPK mRNA (Table S1), and a puromycin resistance gene for cell selection was used.

To generate the pSBtet-shAMPK-BP construct, two initial constructs were initially obtained. MSCV p2GM AMPK alpha2hp1 alpha 1hp1 was a gift from Russell Jones (Addgene plasmid #89492).^47^ pSBtet-BP was a gift from Eric Kowarz (Addgene plasmid # 60496; http://n2t.net/addgene:60496; RRID:Addgene_60496).^62^ pCMV(CAT)T7-SB100 was a gift from Zsuzsanna Izsvak (Addgene plasmid # 34879; http://n2t.net/addgene:34879; RRID:Addgene_34879).^63^ The shRNA scaffold and AMPK shRNA sequence from MSCV p2GM AMPK alpha2hp1 alpha 1hp1 (Table S1) was cloned into pSBtet-BP to replace the luciferase gene (construct generated by Biobasic, Markham, ON, Canada) to generate pSBtet-shAMPK-BP.

MDA-MB-231 cells were cultured to confluence before transfection with FuGENE Transfection Reagent (Promega, E2311) along with pSBtet-shAMPK-BP and pCMV(CAT)T7-SB100. Following transfection, cells were washed with PBS and allowed to recover for 24 h in culture medium. Cells were then dissociated and cultured in a new dish for 2 weeks in culture medium containing 2 μg/mL puromycin. Selected colonies were pooled and FACS sorted based on blue fluorescent protein (BFP) expression (expressed constitutively by the original pSBtet-BP and newly generated pSBtet-shAMPK-BP plasmids). Using FACS, the cells with the greatest BFP intensity (top 30%) of the population was selected and subsequently cultured in 2 mg/mL puromycin containing RPMI media.

#### Microbubbles

DEFINITY microbubbles (Lantheus Medical Imaging Inc., Saint-Laurent, QC) were activated using a Vialmix for 45 seconds according to the manufacturer’s protocol. Microbubbles were used within 1 hour of activation. A 180 μL volume of activated microbubbles was diluted in 570 μL of room temperature PBS to a total volume of 750 μL. The mixture was resuspended by hand agitation and injected into the treatment vessel immediately prior to US exposure, giving a concentration of 10 μL of microbubbles per mL of media.

#### Ultrasound treatment

Previous work in our lab using this ultrasound setup had determined that these parameters are sufficient to induce biological effects in MDA-MB-231, ARPE19, and other adherent cells.^14^^,^^15^^,^^19^ Importantly, in each case, these experiments resulted in reversible sonoporation and a significant portion of the cells remaining viable following USMB treatment. As such, this previous characterization allowed us to gage that these parameters were ideal to study the contribution of stress responsible pathways such as AMPK to the survival of cells that undergo reversible sonoporation. The study design we used is outlined in Figure 1. Briefly, a six-well plate with adherent cells was filled with 16 mL of media and then exposed to an ultrasound wave at 500 kHz pulse centre frequency. We used a single element flat transducer with 32 mm element diameter focused at 85 mm and a −6 dB beam width of 31mm at the focal point (IL0509GP, Valpey-Fisher Inc. Hopkinton, MA, USA), 690 kPa peak negative pressure, 32 μs pulse duration (16 cycle tone burst) at 1 kHz pulse repetition frequency corresponding to 3.2% duty cycle, for 60 seconds in the presence of prepared microbubbles. An arbitrary waveform generator connected to a power amplifier (AG series Amplifier, T&C power conversion Inc., NY) transmitted electrical signal parameters to the ultrasound transducer.

#### Immunofluorescence staining

Cells were seeded onto coverslips in growth medium. Following treatment, cells were washed 2X with PBS (with Mg^2+^ and Ca^2+^) and then immediately placed on ice and fixed with 4% paraformaldehyde for 10 min, quenched with glycine for 5 min, and permeabilized with 0.1% triton X-100 and glycine solution for 5 min. Cell samples were subject to blocking using SuperBlock (ThermoFisher Scientific, cat#37515) according to the manufacturer’s protocol. Cells were stained with anti-Phospho-Acetyl-CoA Carboxylase (Cell Signaling. #11818) using a 1:400 dilution in a PBS supplemented with 1% BSA (1% BSA-PBS) for 1 h at room temperature by the inverted drop technique. Cells were washed over ten times with PBS, then stained with AffiniPure Goat anti-rabbit Alexa Fluor488-conjugated antibody (1:1000 dilution in 1% BSA-PBS) (Jackson ImmunoResearch Laboratories Inc. #111-545-144) for 1 h at room temperature in the dark. Cells were washed over ten times with PBS and mounted onto coverslips with Dako Fluorescent Mounting Medium (Agilent Technology #S3023). The slides were incubated at room temperature overnight to solidify before being stored in −20°C storage until visualization.

#### Epifluorescence microscopy and image analysis

All samples were visualized on an inverted microscope by widefield epifluorescence microscopy (Olympus IX83 epifluorescence microscope, Hamamatsu ORCA FLASH4.0C11440-22CU camera, run by Olympus cellSens software). For each experiment, randomly chosen fields were selected for acquisition of pS79-ACC signal with a 40× magnification using 100 ms exposure time. Mean fluorescence intensity (Figures 1B, 3E, and 3F) was determined using ImageJ software (National Institutes of Health, Bethesda, MD).^64^ Briefly, a cell outline was determined manually by tracing the cell boarder, followed by measurement of the mean fluorescence intensity of the relevant channel in each cell. The non-specific signal, determined similarly in cells subjected to immunofluorescence labeling without primary antibody or fluorescent dye, was subtracted from all values. This mean fluorescence intensity signal was individually measured for >100 cells per condition. Fluorescent intensity was normalized to untreated control and expressed as fold change. Ordinary one-way ANOVA with Tukey’s multiple comparisons test was performed for the conditions.

#### Cell lysis and western blots

Cells were washed twice with cold PBS and lysed with a cell scraper in Laemmli sample buffer supplemented with protease inhibitors (cOmplete™ EDTA-free Protease Inhibitor Cocktail, Roche, cat#1187358001), sodium orthovanadate (1 mM), okadaic acid (1 μM), bromophenol blue (0.004%), and 2-mercaptoethanol (10%). The protein lysates were heated to 65°C and passed through a 27.5 G needle. The lysates were separated on mini-PROTEAN TGX precast gels (Bio-Rad), and transferred onto FluroTrans PVDF (cat#PVM020C-099, PALL Life Sciences) using wet transfer method. Selected primary and secondary antibodies were used as indicated. The immunoreactive bands were developed using Luminata Crescendo Western HRP substrate (cat#WBLUR0100, Millipore Sigma) and detected using a ChemiDoc Touch imaging system (Bio-Rad). Typical exposure times varied between 1-60s and were selected to ensure that signal was not saturated at any pixel. Images were quantified using ImageJ software (National Institutes of Health, Bethesda, MD)^64^ by signal integration in an area corresponding to the appropriate lane and band for each condition.

#### GC-MS for steady state metabolite analysis

Cells were counted via automated cell counting method and 4.50x10^5^ cells were seeded in triplicates (n = 3) in 6-well plates 24 hours prior to treatment. Treated cells were placed on ice and washed twice with ice-cold saline solution (0.9% NaCl). Subsequently, the cells were scraped from the plate while placed on dry ice in 300 μL of 80% methanol chilled to −20°C. The extracts were collected and transferred to prechilled tubes. To ensure complete collection of the material, 300 μL more of 80% methanol was added to the leftover cells in the wells, collected and added to the previously collected 300 μL fraction. Cell extracts were sonicated and the supernatants were extracted via centrifugation. After the supernatants were transferred into Eppendorf tubes, 750 ng of myristic acid-D27 was added to each sample to serve as an internal standard. The supernatants were dried overnight via CentriVap at 4°C (Labconco). The dried residues were dissolved in 30 μL of pyridine containing methoxyamine-HCl (10 mg/mL) (MilliporeSigma). Samples were then incubated for 30 min at 70°C. Afterwards, 70 μL of N-tert-Butyldimethylsilyl-N-methyltrifluoroacetamide (MTBSTFA) was added to the sample mixtures and incubated for 1 h at 70°C. 1 μL of each sample mixture was used for GC–MS analysis. The GC-MS machine and software were obtained from Agilent. Data analyses were performed on ChemStation and MassHunter software (Agilent). We quantified the peak area for each metabolite based on known ion peaks, and normalized this to sample protein content and to an internal loading standard Myristic D-27. Individual metabolite values were averaged and normalized to untreated controls to determine fold changes for each metabolite compared to untreated controls. The data is shown as “Relative levels after USMB” for each metabolite, which is calculated for each metabolite as ([USMB-treated]-[control])/[control].

#### ATP assay

Luminescent ATP detection assay kit (abcam, ab113849) was used to quantify ATP according to manufacturer’s protocol. Briefly, cells seeded in a six-well plate were lysed with 200 μL of the provided detergent and equal volume of substrate solution was added before luminescence detection on a BioTek Synergy HTX Multi-Mode Reader. Results were normalized to the total protein content for the respective treatment as determined though the BCA assay (ThermoFisher, cat#23227). The results were expressed as fold change (means ± SD) over untreated controls. Ordinary one-way ANOVA followed by Tukey’s multiple comparisons test was performed.

#### Cell counting analysis

Treated cells were washed with PBS and then dissociated with 200 μL Trypsin-EDTA (Thermofisher, cat#25200065). 200 μL of complete culture media was added to end the trypsinization reaction. 10 μL of cells was collected in a tube and mixed with 10 μL of 0.4% trypan blue. Cells were counted with a Countess II FL (ThermoFisher Scientific). Three technical replicates for each condition were recorded. Results were averaged and normalized to untreated starting conditions. The results were expressed as fold change of cell number (mean ± SD) over initial untreated control. Two-way ANOVA followed by Tukey’s multiple comparisons test was performed.

#### Cytotoxic assay and cell confluency measurement using the incucyte

Cell death was assessed using CellTox Green Cytotoxicity Assay reagent (Promega G8741). 1 μL of the dye was diluted in 10 mL of media, and this mixture was added to the cells 5 min after USMB treatment. Cytotoxicity and viability were measured using an Incucyte S3 Live-Cell Analysis System (Sartorius). Images were acquired at regular intervals under 10× magnification using brightfield phase contrast and 100ms exposure of 460 nm excitation, 524 nm emission for green fluorescence. Analysis was performed with Incucyte Plategraph for CellTox positive cells and phase confluence. Two-way ANOVA followed by Tukey’s multiple comparisons test was performed.

### Quantification and statistical analysis

All statistical analysis was conducted using GraphPad Prism 8.0.2. Data obtained for GC-MS is representative for at 3 technical replicates of 3 independent experiments and is represented as the mean ± SD. Statistical analysis was performed by unpaired t-tests (comparing control vs USMB-treated samples) for each metabolite. Data obtained from Western Blots is representative for at least 3 independent experiments. Data obtained from IF experiments is representative for 3 independent experiments or as indicated and is represented as the mean ± SD. Statistical analysis was performed by one-way ANOVA followed by Tukey’s multiple comparisons test. Data obtained using the Incucyte S3 Live-Cell Analysis System is representative for at least 3 independent experiments, analyzed as the mean of at least 25 fields of view, and represented as mean ± SEM. Statistical analysis was performed by two-way ANOVA with Sidak’s multiple comparison. The significance of the differences between groups was analyzed as depicted in the respective figure panel and method. P values <0.05 were considered statistically significant. The specific p value is depicted in the representative figure panel.

## Data Availability

• All data reported in this paper will be shared by the lead contact upon request. • This paper does not report original code. • Any additional information required to reanalyze the data reported in this paper is available from the lead contact upon request.
