# Supplementary material for: AMPK is required for recovery from metabolic stress induced by ultrasound microbubble treatment
Source: iScience. 2022 Dec 28;26(2):105883. doi: 10.1016/j.isci.2022.105883 (PMC9845798; doi:10.1016/j.isci.2022.105883)
Supplement: Document S1. Table S1 [file mmc1.pdf]

iScience, Volume *26*

Supplemental information

AMPK is required for recovery from metabolic stress induced by ultrasound microbubble treatment

Louis Lo, Oro Uchenunu, Roberto J. Botelho, Costin N. Antonescu, and Raffi Karshafian

Supplemental Table 1. Sequences used for siRNA and shRNA. Shown in red is the AMPK targeting sequence within the shRNA scaffold, related to STAR Methods.

**Target**	**Forward (5' to 3')**	**Reverse (5' to 3')**	**Application**
AMPK α1/2	GCACCUUCGGCAAAGUGAAUU	UUCACUUUGCCGAA<br>GGUGCUU	siRNA
non-targeting (control)	CGUACUGCUUCGGAUACGGU<br>U	CCGUCUCGCAAGCA<br>GUACGUU	siRNA
pSBtet - shRNA scaffold and AMPK shRNA sequence (red)	Ccgcagcagcagcatttaaatggccgcaa<br>gccttgttaagtgctcgcttcggcagcacatat<br>actatgttgaatgaggcttcagtactttacaga<br>atcgttgcctgcacatcttggaaacacttgctg<br>ggattacttcttcaggttaacccaacagaagg<br>ctcgagaaggtatattgctgttgacagtgagc<br>gcggccataaagtggcagttaaatagtgaa<br>gccacagatgtatttaactgccactttatggcc<br>ttgcctactgcctcggaattcaaggggctactt<br>taggagcaattatcttgtttactaaaactgaat<br>accttgctatctctttgatacatttttacaaagct<br>gaattaaaatggtataaattaaatcactttttc<br>aattggaagactaatgcgtttaaatggccgc<br>aagccttgttaagtgctcgcttcggcagcaca<br>tatactatgttgaatgaggcttcagtactttaca<br>gaatcgttgcctgcacatcttggaaacacttg<br>ctgggattacttcttcaggttaacccaacaga<br>aggctcgagaaggtatattgctgttgacagtg<br>agcgcggaggagagctatttgattattagtga<br>agccacagatgtaataatcaaatagctctcct<br>ccatgcctactgcctcggaattcaaggggct<br>actttaggagcaattatcttgtttactaaaactg<br>aataccttgctatctctttgatacatttttacaaa<br>gctgaattaaaatggtataaattaaatcactttt<br>ttcaattggaagacta		Sleeping Beauty Transposon System
